# Beating stress: Evidence for recalibration of word stress perception

**DOI:** 10.3758/s13414-025-03088-5

**Published:** 2025-05-20

**Authors:** Ronny Bujok, David Peeters, Antje S. Meyer, Hans Rutger Bosker

**Affiliations:** 1 Max Planck Institute for Psycholinguistics, Nijmegen, The Netherlands; 2 International Max Planck Research School for Language Sciences, MPI for Psycholinguistics, Max Planck Society, Nijmegen, The Netherlands; 3 Department of Communication and Cognition, TiCC, Tilburg University, Tilburg, The Netherlands; 4 Donders Institute for Brain, Cognition and Behaviour, Radboud University, Nijmegen, The Netherlands

**Keywords:** Speech perception, Adaptation and aftereffects, Multisensory processing

## Abstract

Speech is inherently variable, requiring listeners to apply adaptation mechanisms to deal with the variability. A proposed perceptual adaptation mechanism is recalibration, whereby listeners learn to adjust cognitive representations of speech sounds based on disambiguating contextual information. Most studies on the role of recalibration in speech perception have focused on variability in particular speech segments (e.g., consonants/vowels), and speech has mostly been studied with a focus on talking heads. However, speech is often accompanied by visual bodily signals like hand gestures, and is thus multimodal. Moreover, variability in speech extends beyond segmental aspects alone and also affects prosodic aspects, like lexical stress. We currently do not understand well how listeners adjust their representations of lexical stress patterns to different speakers. In four experiments, we investigated recalibration of lexical stress perception, driven by lexico-orthographical information (Experiment 1) and by manual beat gestures (Experiments 2–4). Across experiments, we observed that these two types of disambiguating information (presented in an audiovisual exposure phase) led listeners to adjust their representations of lexical stress, with lasting consequences for subsequent spoken word recognition (in an audio-only test phase). However, evidence for generalization of this recalibration to new words was only found in the third experiment, suggesting that generalization may be limited. These results highlight that recalibration is a plausible mechanism for suprasegmental speech adaption in everyday communication and show that even the timing of simple hand gestures can have a lasting effect on auditory speech perception.

## Introduction

Speech produced by different speakers can vary a lot in terms of actual realization. The same word can sound very different depending on who is producing it. This variation poses a problem for our speech perception system tasked with accurately determining what is being said. Listeners must adapt to different speakers and their specific ways of producing speech. One of the ways to achieve this is by adjusting perceptual category boundaries (e.g., for individual speech sounds) to accommodate the speaker’s way of speaking (for review, see Ullas et al., 2022). This process is known as *recalibration* (e.g., Bertelson et al., 2003; Norris et al., 2003)*.* Spoken utterances, however, typically combine segmental with suprasegmental information, such as lexical stress patterns and prosodic contours (for review, see Cutler, 2008), which also vary in their realization (e.g., Severijnen et al., 2024; Xie et al., 2021), necessitating some form of adaptation (e.g., recalibration) to suprasegmental variability as well. Critically, in many conversations we can see the speaker, allowing us to use spoken auditory information and visual information, for instance via co-speech hand gestures, to derive what the speaker intends to convey (Holler & Levinson, 2019; Kita & Özyürek, 2003; McNeill, 2008; Wagner et al., 2014). We do not yet fully understand whether and how suprasegmental information is recalibrated, and how in this process spoken and manual sources of information may jointly play a role. This study therefore aimed to test whether listeners can recalibrate their perception of suprasegmental information, specifically lexical stress, when perceiving multimodal messages.

Recalibration is a domain-general perceptual mechanism (e.g., Noppeney, 2021) that serves to achieve perceptual constancy in the perceiver despite variability in the input. It has been observed in color perception (Mitterer & de Ruiter, 2008), auditory spatial localization (Radeau & Bertelson, 1974), audiovisual synchrony perception (Fujisaki et al., 2004; Vroomen, et al., 2004a, 2004b), and spoken word recognition (Bertelson et al., 2003; Norris et al., 2003). In the latter field, it has been proposed that listeners use recalibration to deal with variability in speech. If, for example, someone hears an ambiguous fricative, lying in between an/f/and/s/, they can learn to interpret the ambiguous fricative as either an/f/or an/s/depending on disambiguating information (Norris et al., 2003). For instance, when participants are repeatedly presented with the ambiguous fricative in a lexical context that disambiguates the sound as an/f/(e.g., by hearing it in the word “gira?”), they can learn to categorize the sound as/f/. In contrast, in an/s/-biasing context (e.g., hearing it in the word “platypu?”), they learn to categorize the same sound as/s/. Crucially, studies show that even in a subsequent test phase without any disambiguating lexical information, people still categorize the ambiguous sound as either/f/or/s/depending on the biasing context they had been exposed to earlier (Norris et al., 2003). Recalibration is believed to involve changes in the perceptual boundaries of abstract phoneme representations, such that the initially ambiguous fricative is considered an acceptable token of/f/(after exposure to/gira?/) or/s/(after exposure to/platypu?/) (Kleinschmidt & Jaeger, 2015; Xie et al., 2023).

Studies have found recalibration effects in word recognition driven by various types of disambiguating information, including lexical (Norris et al., 2003), semantic (Jesse, 2019), lexico-orthographic (Bosker, 2022; Keetels et al., 2016), and visual articulatory cues (Bertelson et al., 2003). For instance, when repeatedly exposed to an ambiguous sound between a/b/and a/d/together with a video of the speaker’s face producing either a visual/b/or/d/(i.e., lips closing vs. tongue touching alveolar ridge), participants recalibrated their perception of the ambiguous sound. That is, participants who saw a visual/b/were more likely to perceive the ambiguous sound as a/b/in a later audio-only test phase than participants who had seen a visual/d/. The participants’ perception of the same auditory stimulus changed based on the disambiguating visual information provided earlier (Bertelson et al., 2003). This observation has been taken as evidence for participants forming abstract representations of speech sounds, recalibrating their perception of different cues based on the disambiguating context, and then using the recalibrated representations to comprehend a speaker on following occasions, even in the absence of disambiguating cues.

However, speech can differ between speakers in many more ways than just the segments. Indeed, speech can also differ in its prosodic properties, such as speech rate (Maslowski et al., 2019) and lexical stress (Severijnen et al., 2021). Prosodic information can play a significant role in word recognition. In some languages including Dutch, which is studied here, lexical stress is contrastive, meaning that there are instances where lexical stress is the only cue differentiating two segmentally identical words (e.g., Dutch *VOORnaam* [first name] vs. *voorNAAM* [respectable];/vo:r.na:m/). In these instances, lexical stress is crucial to understand which word the speaker means. Just like segmental variation, the production of lexical stress can vary significantly between speakers (e.g., Severijnen et al., 2024). It can be conveyed by various acoustic cues such as fundamental frequency (F0), duration, and intensity (Rietveld & Heuven, 2009), and different speakers use these cues differently and to varying degrees (Severijnen et al., 2024). Therefore, the variability problem introduced above extends to suprasegmental aspects of speech. Hence, people might benefit from adaptation to different prosodic realizations of speech.

Previous studies have found recalibration of prosodic aspects of speech including lexical tone in Mandarin (Mitterer et al., 2011), sentence-level intonation in English (Kurumada et al., 2012), and vocal affect (Baart & Vroomen, 2018). One study has found that recalibration of lexical stress perception is also possible (Bosker, 2022). Participants in that study listened to ambiguously stressed stimuli from a lexical stress continuum of a single Dutch minimal pair (*CAnon* [canon] and *kaNON* [cannon];/ka:.nɔn/). One group of participants listened to the ambiguous stimuli with a concurrent orthographic word form presented on a computer screen indicating stress on the first syllable (strong–weak; SW, e.g., *CAnon*). Another group heard the same ambiguous speech while seeing an orthographic word form indicating stress on the second syllable (weak-strong; WS, e.g., *kaNON*). It was observed that participants learned to associate the ambiguous acoustic properties of the stimuli with either a strong–weak or a weak-strong lexical stress pattern. That is, in a later test phase, they were instructed to categorize words taken from a lexical stress continuum of the same word pair (*CAnon – kaNON*) as either strong–weak or weak-strong. Crucially the test phase was audio-only; that is, no disambiguation by orthographic forms on-screen was provided. The group that had listened to the stimuli while seeing the SW orthographic form on-screen in exposure categorized the entire continuum as more SW-like in the test phase (i.e., gave a higher proportion SW responses) than the group that had listened to the same stimuli while seeing the WS orthographic form on-screen in exposure (Bosker, 2022).

Moreover, the same study also found evidence for generalization of the recalibration acquired during exposure to novel word items at test. That is, when new participants were presented with a segmentally different stress continuum (*SERvisch* [Serbian] – *serVIES* [crockery]) in exposure, they too perceived the same *CAnon – kaNON* continuum at test as either more SW-like or WS-like depending on whether the disambiguating orthographic form in exposure indicated SW or WS stress, respectively. This could indicate that participants do not only learn stress patterns on a word-by-word basis, but that their changed perception of lexical stress can be generalized and applied to different words.

Importantly, however, Bosker (2022) used highly artificial speech continua in the experiment. Duration and intensity for each syllable were set to averaged, ambiguous values. Moreover, the original F0 (fundamental frequency) contours of the recorded speech were removed and replaced by artificial linear downward slopes for each syllable with its mean F0 height varying across the continuum. That is, the SW word had a relatively high mean F0 on the first syllable and a relatively low mean F0 on the second syllable. In contrast, the WS word had a relatively low mean F0 on the first syllable and a relatively high mean F0 on the second syllable. For the ambiguous steps, the mean F0 for the syllables was gradually lowered or raised to create the continuum. Most critically, this artificial F0 manipulation was then applied to *both word pairs* (i.e., same F0 values and contours in the *canon-kanon* continuum as in the S*ervisch-servies* continuum). Hence, participants in the Bosker study (2022) demonstrated evidence of generalizing their recalibration effect to a segmentally different continuum with different ambiguous durations and intensities, but identical step-like F0 contours. This means that the generalization effect found in Bosker (2022) does not necessarily reflect an adaptation of abstract representations of stress patterns but could also reflect an adaptation to specific F0 values. Hence, one goal of the present study was to assess whether recalibration and generalization are also possible with more naturalistic F0 contours and thus more acoustic distance between the words.

The second and most central goal of the current study was to assess whether listeners can use *visual* information, such as manual beat gestures (see below), to recalibrate perception of suprasegmental aspects of speech such as lexical stress. While the production of lexical stress is less clearly associated with visual articulatory cues than certain speech segments (e.g., salient mouth closing when producing a/b/), it nevertheless has visual correlates such as the typically wider and longer mouth opening on stressed syllables (Scarborough et al., 2009). These articulatory cues are visible and used by participants to categorize “talking faces” (i.e., muted videos) producing different stress patterns (Bujok et al., 2025; Jesse & McQueen, 2014; Scarborough et al., 2009). However, when presented with audiovisual (AV) stimuli, the same visual articulatory information indicating stress does not seem to strongly affect audiovisual perception (Bujok et al., 2025). That is, the same sound, paired with a face articulating stress on either the first or the second syllable, was perceived similarly in a previous study (Bujok et al., 2025). Similar findings have also been reported regarding Mandarin tone perception (Han et al., 2020), where available visual cues are not being used in audiovisual perception. Therefore, it is unlikely that facial articulatory cues to stress, which do not even appear to be used in on-line audiovisual stress perception, could drive recalibration.

In contrast, other visual cues could be used to recalibrate the perception of lexical stress. Hand gestures are commonly produced in face-to-face conversations and have been shown to affect spoken word recognition, particularly in noisy settings (Drijvers & Özyürek, 2017). One particular group of hand gestures, the so-called beat gestures, mainly defined as simple bi-phasic up-and-down movements of the hands, tend to align with acoustically prominent parts of utterances (Krahmer & Swerts, 2007). Specifically, the point of maximum extension of the beat gesture, the so-called apex, is strongly temporally related with pitch accent (Leonard & Cummins, 2011) and affects the acoustic realization of the pitch accent as well (Krahmer & Swerts, 2007; Pouw et al., 2020; Swerts & Krahmer, 2007), making the accented utterance even more prominent (Krahmer & Swerts, 2007). Beat gestures have been found to help listeners focus their attention on important information (Biau & Soto-Faraco, 2013, 2015) and to increase processing of the focused words (Dimitrova et al., 2016). Moreover, beat gestures boost memory recall of the words they are aligned with (Kushch & Prieto, 2016). However, we do not know whether listeners make use of the information provided by beat gestures for suprasegmental recalibration purposes.

On the word level, the apex of a beat gesture is usually temporally aligned to the F0 peak of a stressed syllable in free-stress languages like English and Dutch (Leonard & Cummins, 2011; Shattuck-Hufnagel & Ren, 2018). As such, listeners can take advantage of this close temporal link and use it in lexical stress perception. That is, people are more likely to perceive stress on a syllable when a beat gesture is aligned to it. For instance, when Dutch participants hear tokens from a lexical stress continuum of/ka:.nɔn/(ranging from *CAnon* to *kaNON*) with a beat gesture on the first syllable, they are more likely to report perceiving *CAnon* rather than *kaNON* (Bosker & Peeters, 2021; Bujok et al., 2025). This effect is robust across several different word pairs and different phonetic continua (Bosker & Peeters, 2021; Bujok et al., 2025), and has been demonstrated in Dutch (Bosker & Peeters, 2021; Bujok et al., 2025) and Spanish (Rohrer et al., 2020). Moreover, it has also been reliably found both in a short 10-min study (Maran & Bosker, 2024) as well as across multiple sessions more than 1.5 years apart (Cos et al., 2024).

Given this effect, we hypothesized that the temporal alignment of beat gestures with speech could be a cue for recalibration of lexical stress, in analogy to how a talking face can recalibrate the perception of a/b/–/d/continuum (Bertelson et al., 2003). Essentially, we asked whether the effect of beat gestures goes beyond the immediate effect of disambiguating an ambiguously stressed word (Bosker & Peeters, 2021) and would lead to lasting changes in speech perception. Finding evidence for a recalibration effect driven by beat gestures would extend current models of recalibration (Kleinschmidt & Jaeger, 2015; Xie et al., 2023) to include visual cues beyond articulation (Bertelson et al., 2003) and lexico-orthographical information (Bosker, 2022). Such evidence would be consistent with multimodal frameworks of spoken language comprehension (Holler & Levinson, 2019; Özyürek, 2014).

In four behavioral experiments, we tested participants’ ability to recalibrate their perception of lexical stress in a specific word, as well as their ability to generalize recalibration to a novel item. In the first three experiments we used a similar experimental design to that of Bosker (2022), which was based on classic experiments on lexical recalibration (Norris et al., 2003). We first targeted recalibration guided by disambiguating written word forms (Experiment 1) and then targeted – for the first time – recalibration guided by beat gestures (Experiments 2–4). Thus, in the first experiment, we adopted a paradigm similar to Bosker (2022), testing recalibration of the perception of lexical stress by written information. Critically, we used different stimuli, with more naturalistic phonetic stress continua based on the original F0 contours. Consequently, the F0 contours for the two minimal word pairs (used to test generalization of recalibration to new words) were distinct. This arguably makes finding evidence for generalization more difficult but it does better reflect naturalistic spoken communication, where every F0 contour is unique. In the second and third experiments, we used the same auditory stimuli and a similar experimental paradigm but tested whether the recalibration of lexical stress perception could be driven by the temporal alignment between spoken words and visual beat gestures. Finally, the fourth experiment used an experimental design inspired by previous studies on visually guided recalibration (see Bertelson et al., 2003) to test for the presence of recalibration in the absence of other potential adaptation mechanisms.

## Experiment 1: Recalibration driven by words on-screen

The first experiment was a conceptual replication of Bosker (2022), but with different stimuli to test whether recalibration effects can be found with more naturalistic stimuli. We expected to replicate the original recalibration findings in the Recalibration Condition (i.e., when participants are tested on the same word pair they were exposed to; note this was labeled the *Segmental Overlap* condition in Bosker, 2022). However, given our more variable and naturalistic phonetic continua, we were not certain about the generalization of the effect to different words. If the generalization effect found by Bosker (2022) was at least in part driven by the artificial and identical F0 continua between the word pairs, we should not find a generalization effect with more naturalistic stimuli. On the other hand, finding a generalization effect here would provide strong evidence that listeners are in fact able to generalize recalibration to different words with similar, but not identical, stress cues.

### Method

#### Participants

All participants tested in this study gave informed consent as approved by the Ethics Committee of the Social Sciences Department of Radboud University (project code: ECSW-2019–019). Only participants who reported no hearing or language deficit and normal or corrected-to-normal vision participated. Participants were financially compensated for their participation. We recruited unique samples of participants for each experiment. For Experiment 1 we tested 72 native speakers of Dutch (59 female, 13 male), recruited from the Max Planck Institute for Psycholinguistics participant database. Their median age was 24 years (SD = 3.67, range = 18–36 years).

#### Materials

Materials for this experiment were adopted from previous experiments (Bujok et al., 2025). Two disyllabic, segmentally identical minimal stress pairs of Dutch, which only differed in the position of lexical stress, were chosen (*CAnon* [canon] vs. *kaNON* [cannon]; *VOORnaam* [first name] vs. *voorNAAM* [respectable]; capitals indicate lexical stress). We recorded high-definition videos of a male native speaker of Dutch (i.e., the last author), while he was sitting down, producing these words naturally without any manual gesture. The audio sampling rate was 48 kHz.

**Fig. 1:**
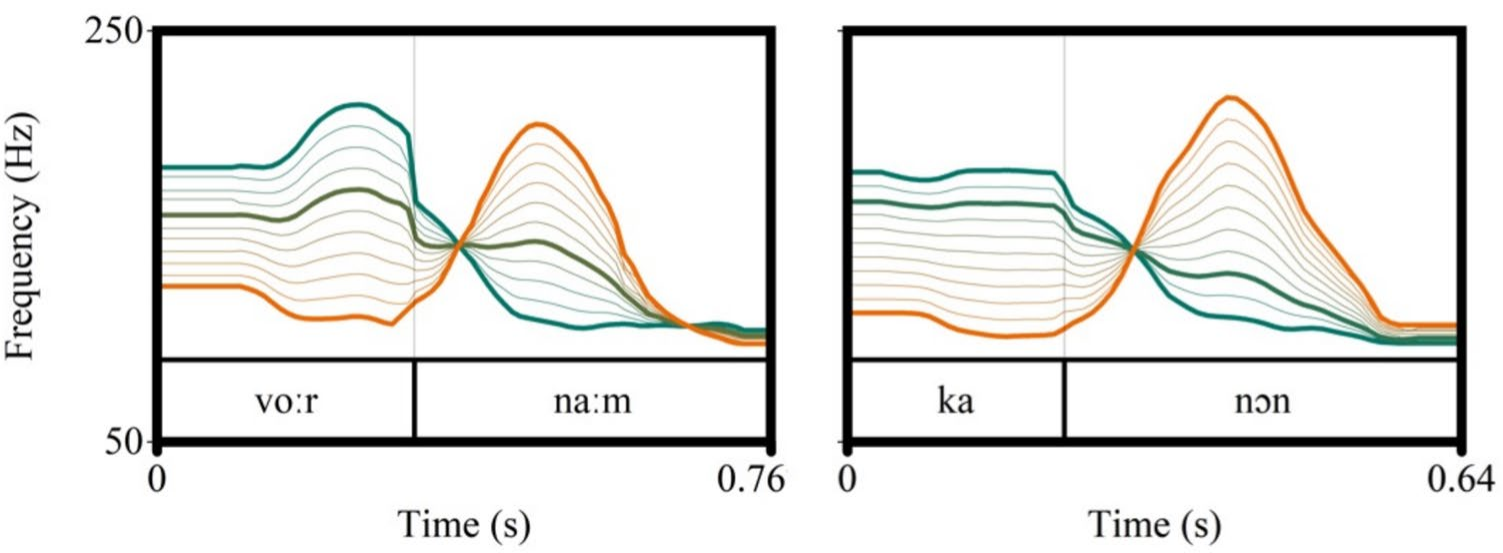
Visualization of the F0 stress manipulation for/vo:r.na:m/(**left panel**) and/ka:.nɔn/(**right panel**): F0 contours were interpolated in 11 steps to go from SW (green) to WS (orange). Five manipulated steps were selected and presented with the original SW and WS recordings as a perceptual seven-step continuum. The extremes of the continua and the perceptually most ambiguous step are highlighted in bold. *Note:* The most ambiguous step was determined as most ambiguous in a perceptual pretest and labeled Step 4 in the final seven-step continuum (for pretest results see Fig. S5 in the Online Supplementary Materials, https://osf.io/s3p6a/). Steps 1 and 2 of the final seven-step/ka:.nɔn/continuum had similar F0 contours, but were still acoustically different because Step 1 also contained original duration and intensity stress cues, and Step 2 did not

A lexical stress continuum was created by measuring the F0 contours of the original recordings and then linearly interpolating between the contours in 11 steps (ranging from the original SW recording to the original WS recording, see Fig. 1). For each pair we set the duration and intensity of each syllable to the average, ambiguous values (i.e., midway between stressed/unstressed) based on the original recordings. Duration and intensity were kept constant at these ambiguous values across the entire continuum (for overview of duration and intensity values see Table S1 in the Online Supplementary Materials (OSM) on the Open Science Framework at: https://osf.io/s3p6a/). The interpolated F0 contours were then applied to the SW token using PSOLA in Praat (Boersma, 2006). Note that this contrasts with the continuum manipulation in Bosker (2022), where F0 contours on either syllable always involved linear downward slopes, only varying in mean F0 height, removing any sign of the original contours. Another consequence of these artificial manipulations in Bosker (2022) was that both continua were identical with regard to F0. In contrast, our more naturalistic contour interpolation method entailed every manipulated stimulus having a unique F0 contour.

The manipulated 11-step continua were presented to ten participants (who did not participate in any of the experiments) in a pretest in a two-alternative forced-choice (2 AFC) task, where they had to categorize the words as either SW or WS. Based on the categorization results, we selected five perceptually ambiguous steps for each pair, which ranged between ~ 80% and ~ 20% SW responses, to create a perceptual continuum. We have provided the pretest categorization data in the OSM (see Fig. S5, https://osf.io/s3p6a/). The five selected steps were then combined with the original, unmanipulated tokens resulting in a seven-step continuum. Step 1 thus refers to the original SW token and Step 7 to the original WS token. This meant that Steps 1 and 7 contained original duration, intensity, and F0 cues indicating stress. In contrast, the middle five steps of each continuum only cued stress through F0, as duration and intensity were set to constant and ambiguous values (i.e., midway between stressed/unstressed). Note that the pretest unexpectedly demonstrated that Step 3 from the 11-step/ka:.nɔn/continuum was perceptually the most ambiguous. Therefore, this Step 3 was mapped onto the middle Step 4 of the final seven-step continuum. Similarly, Step 2 was mapped onto Step 3, Step 1 onto Step 2, and the original SW recording onto Step 1. Consequently, Step 1 and Step 2 of this final seven-step/ka:.nɔn/continuum had a similar F0 contour (i.e., original F0 cues; see Fig. 1), but the steps were still acoustically different because Step 1 also had original duration and intensity cues indicating SW stress, while Step 2 did not. The five steps in between (Steps 2–6), with varying degrees of ambiguity, will be referred to as *ambiguous steps*. The middle step (Step 4) was the most ambiguous, lying closest to 50% SW categorization responses.

#### Design and procedure

Data for all experiments reported here were collected online using the Gorilla Experiment Builder (http://gorilla.sc) (Anwyl-Irvine et al., 2020). Participants had to complete a headphone screening prior to the experiments, to ensure usage of high quality headphones (based on Huggins Pitch, see Milne et al., 2021). We adopted the design used by Bosker (2022), consisting of two phases: an exposure phase and a test phase. In the exposure phase, participants were assigned to one of two conditions: The Recalibration Condition or the Generalization Condition. These conditions were identical in their procedure, but differed in the items presented during exposure (see Table 1). Note that the Generalization Condition still tested recalibration, albeit generalization of recalibration to segmentally different words.

**Table 1:** Overview of the design of Experiment 1: conditions and stimuli presented

Condition	Group bias	Exposure (AV)		Test (A-only)
		Audio	Video	
Recalibration	SW bias	amb. /ka:.nɔn/ (Step 4)	*CAnon*	/ka:.nɔn/continuum (Steps 2–6)
		WS/ka:.nɔn/(Step 7)	*kaNON*	
	WS bias	amb./ka:.nɔn/(Step 4)	*kaNON*	
		SW/ka:.nɔn/(Step 1)	*CAnon*	
Generalization	SW bias	amb./vo:r.na:m/(Step 4)	*VOORnaam*	
		WS/vo:r.na:m/(Step 7)	*voorNAAM*	
	WS bias	amb./vo:r.na:m/(Step 4)	*voorNAAM*	
		SW/vo:r.na:m/(Step 1)	*VOORnaam*	

In the Recalibration Condition, participants were randomly divided into two counter-balanced groups (18 participants per group). In the SW-Bias group, on half of the trials, participants were presented with an audio recording of a speaker producing a clear token of the word *kaNON* (i.e., stress on second syllable; WS; Step 7 from the seven-step continuum) while being presented with the orthographic form “kaNON” on screen (with instructions that capitalized letters indicated stress). Additionally, on other trials, they were presented with an acoustically ambiguous auditory token (Step 4 from the seven-step continuum), which was disambiguated by the orthographic form “CAnon” with stress on the first syllable appearing on screen. Consequently, the SW-Bias group was predicted to learn that the talker produced ambiguous auditory stress cues associated with an SW prosodic pattern. In contrast, the other group (WS-Bias) was presented with audio recordings of the speaker producing a clear token of the word *CAnon* (i.e., stress on first syllable; SW) while seeing “CAnon” on-screen. Moreover, they were also presented with the acoustically ambiguous auditory token, critically together with the written word “kaNON” on-screen. This group was thus biased to associate the ambiguous stress cues with a WS prosodic pattern. The clear audio trials and the ambiguous audio trials were presented 24 times each, resulting in 48 exposure trials. Participants passively listened to all the stimuli (interstimulus interval: 600 ms, static fixation cross). Then they moved on to the test phase described below.

In the Generalization Condition, the design and procedure of the exposure phase was similar to the Recalibration Condition. However, during the exposure phase, participants in the Generalization Condition were presented with a different item pair. Specifically, the SW-Bias group received a clear auditory *voorNAAM* with the congruent orthographic form “voorNAAM” with stress on the second syllable, and an ambiguous auditory token from the *VOORnaam – voorNAAM* continuum (Step 4) with a disambiguating orthographic form “VOORnaam.” Conversely, the WS-Bias group got a clear *VOORnaam* with congruent “VOORnaam” on-screen, and the ambiguous token (Step 4) with a disambiguating written word “voorNAAM” on-screen.

All participants received the same test phase. That is, they were tested on the same manipulated F0 continuum made up of the five ambiguous steps from the *CAnon – kaNON* continuum (i.e., Steps 2–6) in a 2 AFC task. Hence participants from the Recalibration Condition were tested on the word pair they had been exposed to, whereas participants from the Generalization Condition were tested on a different word pair than they had been exposed to. Each step was presented 15 times, equaling a total of 75 trials presented in random order. After stimulus offset, two response options were shown, one on either side of the screen. Participants were asked to categorize what they heard as corresponding either to *CAnon* (SW) or *kaNON* (WS) by pressing the left (“Z”) or right (“M”) button on their keyboard, corresponding to the left and right word on the screen respectively. The position of SW and WS words on-screen was counter-balanced across participants. Participants were given a 4,000-ms time limit to respond.

### Results

We removed all trials where participants failed to give a response (*n* = 14, 0.25% of all observations). We analyzed our data with Generalized Linear Mixed Models (GLMMs) using the lme4 library (Bates et al., 2015) in R (R Core Team, 2021). The independent variable was the participants’ Categorization Response (SW coded as 1, *CAnon*; WS coded as 0; *kaNON*). Fixed effects included Continuum Step (continuous, z-scored), Group Bias (categorical, deviance coded SW as 0.5 and WS as −0.5), Condition (categorical, deviance coded Recalibration as −0.5 and Generalization as 0.5) and the interactions of Condition with the other fixed effects. Additionally, the model included random effects for Participants, as well as maximal random slopes for Continuum Step, Group Bias, and Condition (*Response* ~ Condition*(Step + *Group Bias)* + *(1* + *Condition* + *Step* + *Group Bias|Participant_ID)*). All data and code are publicly available on the Open Science Framework (https://osf.io/s3p6a/).

**Fig. 2:**
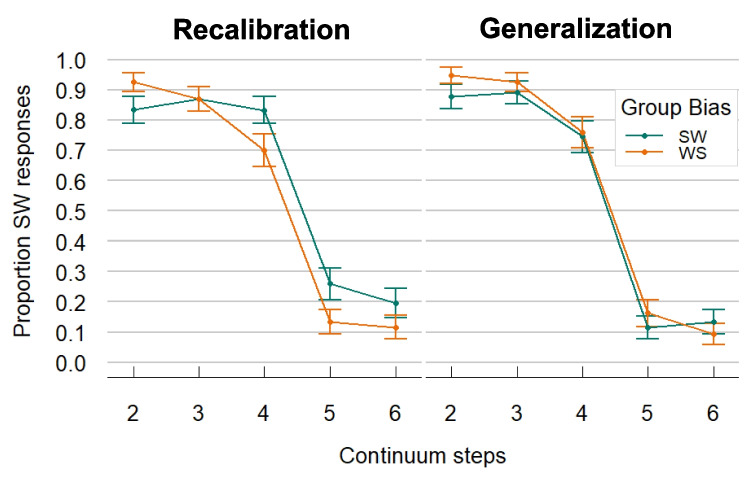
Results from Experiment 1: Comparison of the audio-only test results in the Recalibration and Generalization Condition. The proportion of SW responses (i.e., stress on the first syllable) generally decreases as auditory Continuum Step increases (i.e., sounding more WS-like, stress on the second syllable). Different audiovisual (AV) exposure to disambiguating orthographic word forms in the two groups (SW-Bias vs. WS-Bias) changed responses to the audio only (A-only) test continuum in the Recalibration Condition, as can be seen by the separation of the two lines in the left panel. That is, participants who were exposed to ambiguous/ka:.nɔn/in exposure, paired with orthographic form “Canon,” generally perceived the continuum as more SW-like than participants who were exposed to the same ambiguous tokens of/ka:.nɔn/paired with”kaNON.” However, there was no main effect of Group Bias in the Generalization Condition. Note: Continuum steps go from 2–6, as participants were only tested on the five ambiguous tokens of the seven-step continuum. SW = strong–weak, stress on first syllable; WS = weak-strong, stress on second syllable. Error bars indicate the 95% confidence interval

The model showed a significant Intercept, demonstrating an overall bias to give slightly more SW than WS responses (mean proportion of SW responses = 0.57; *β* in logit space = 0.863, *SE* = 0.113, *z* = 7.649, *p* < 0.001). Continuum Step was also significant (*β* = −3.13, *SE* = 0.278, *z* = −11.272, *p* < 0.001), indicating that participants’ proportions of SW responses decreased with increasing Continuum Steps. Condition (*β* = 0.08, *SE* = 0.222, *z* = 0.36, *p* = 0.72) and its interaction with Continuum Step (*β* = −0.5, *SE* = 0.539, *z* = −0.927, *p* = 0.354) were not significant suggesting similar response patterns across the Recalibration and Generalization Conditions. Most critically, the predictor Group Bias did not have a main effect on the responses (*β* = −0.18, *SE* = 0.156, *z* = 1.156, *p* = 0.248), but showed an interaction with Condition (*β* = −0.745, *SE* = 0.306, *z* = −2.436, *p* = 0.015), meaning that the effect of Group Bias was stronger in the Recalibration Condition than the Generalization Condition (see Fig. 2). In fact, two follow-up models that were run on each condition separately confirmed that the Group Bias effect was significant in the Recalibration Condition (*β* = 0.566, *SE* = 0.247, *z* = 2.286, *p* = 0.022) in the expected direction: the proportion of SW responses was higher in the SW-Bias group compared to the WS-Bias group. In contrast, no effect of Group Bias was found for the Generalization Condition (*β* = −0.215, *SE* = 0.189, *z* = −1.14, *p* = 0.257).

Finally, an exploratory post hoc model on the Recalibration Condition subset, including Time (five batches of 15 consecutive trials) and its interaction with Bias revealed a significant Time*Bias interaction (*β* = −0.273, *SE* = 0.087, *z* = −3.148, *p* = 0.002), meaning that the recalibration effect decreased over time (see Fig. S1 in the OSM).

#### Interim discussion

The current experiment aimed to conceptually replicate the findings from Bosker (2022) using more naturalistic stimuli. That is, we tested whether listeners were able to recalibrate their perception of lexical stress based on disambiguating lexico-orthographic information. In the Recalibration Condition (i.e., when tested on the same word as heard during exposure), participants showed a group-dependent bias in their perception, which was shifted in the direction of the disambiguating information in exposure (e.g., ambiguous audio disambiguated by written “CAnon” leading to more “CAnon” responses).

Post hoc analyses revealed that the recalibration effect decreased over time, which is consistent with previous studies (e.g., Vroomen, van Linden, et al., 2004b). However, Vroomen et al. (2004a) found that the effect could dissipate within six trials. In contrast, we were able to find a consistent effect when we averaged results across 75 trials. This suggests a much slower dissipation of the effect in the present experiment. Reasons for this difference are discussed in the *General discussion*.

In the Recalibration Condition we found a Group Bias not only for the specific most ambiguous token (Step 4) that was presented in the exposure phase, but also for other tokens on the continuum, indicating a certain degree of generalization to novel acoustic tokens of the same word pair. However, participants did not generalize their recalibration to a segmentally different word, contrasting with the findings in Bosker (2022). There were two major differences between our experiment and that of Bosker, both related to the stimuli that were used. First, Bosker created artificial, linear slopes at different mean F0 heights, separately for each syllable, to generate the continua. Second, they applied identical F0 slopes to both items. This manipulation procedure likely facilitated generalization from one word in exposure to a novel item at test, carrying the exact same F0 contour as in exposure. In contrast, we tested for a recalibration effect using more naturalistic continua. By interpolating the original F0 contours of both members of a pair, we created more complex and arguably more natural continua. Not only the height but also the overall shape of the F0 contour cued stress in our stimuli. A consequence of this procedure was that the continua of the two item pairs were acoustically unique and distinct (see Fig. 1). Despite these differences in comparison with Bosker (2022), we also found a recalibration effect. In fact, our data suggest that the perception of lexical stress can recalibrate even with more naturalistic continua than those used by Bosker (2022). However, we did not find a generalization effect. Thus, we caution that the generalization of the recalibration effect to novel words is sensitive to aspects of the stimuli used.

## Experiment 2: Recalibration driven by beat gestures

In Experiment 1 we found that disambiguating orthographic cues can lead to changes in stress perception. However, we rarely encounter speech-disambiguating orthography when conversing with people. In contrast, manual beat gestures are ubiquitous (McClave, 1994) and part of our everyday face-to-face communication (Holler & Levinson, 2019; Hübscher & Prieto, 2019; Kita & Özyürek, 2003). Beat gestures are temporally closely aligned to stressed syllables (e.g., Krahmer & Swerts, 2007; Pouw & Dixon, 2019; Shattuck-Hufnagel & Ren, 2018), which affects online perception of lexical stress (Bosker & Peeters, 2021; Bujok et al., 2025). That is, the alignment of a beat gesture with a syllable makes participants more likely to perceive stress on that syllable. Moreover, beat gestures have been found to redirect attention to a concurrently produced word (Biau & Soto-Faraco, 2013) and are processed and integrated automatically with speech (Kelly et al., 2010). As such, they could be a possible source of disambiguation inducing recalibration of lexical stress. We tested this hypothesis by running Experiment 2, which was similar in design to Experiment 1, but used temporally aligned beat gestures rather than orthographic words as disambiguating information in exposure. Participants in different groups were exposed to videos of a talker producing an ambiguously stressed word with a beat gesture on either the first (SW) or the second syllable (WS). If beat gesture alignment in audiovisual exposure stimuli can be used as a cue to recalibration, participants in the SW and WS groups should be biased to perceive the same audio-only stress continuum in the test phase differently.

### Method

#### Participants

Seventy-two native speakers of Dutch (34 female, 37 male, one gender not reported) were recruited for this experiment through Prolific. Median age was 25 years (SD = 4.9, range = 19–38 years).

#### Materials

For this study we again used stimuli from Bujok et al. (2025). For a detailed description of the audio and phonetic manipulations see the *Materials* section for Experiment 1. Additionally, for Experiment 2 we used video stimuli to test whether visual beat gestures could drive recalibration of lexical stress perception. The same talker from Experiment 1 had been video-recorded producing all four words (*CAnon, kaNON, VOORnaam, voorNAAM*) with a beat gesture. The beat gesture was an up-and-down, forward-rotating movement of the right hand, with the apex (the point of maximal extension) naturally aligned to the stressed syllable. The speaker was sitting in front of a neutral background and framed from the hip up. Videos were recorded at a sampling rate of 50 Hz and cropped to 620 × 620 pixel squares.

**Fig. 3:**
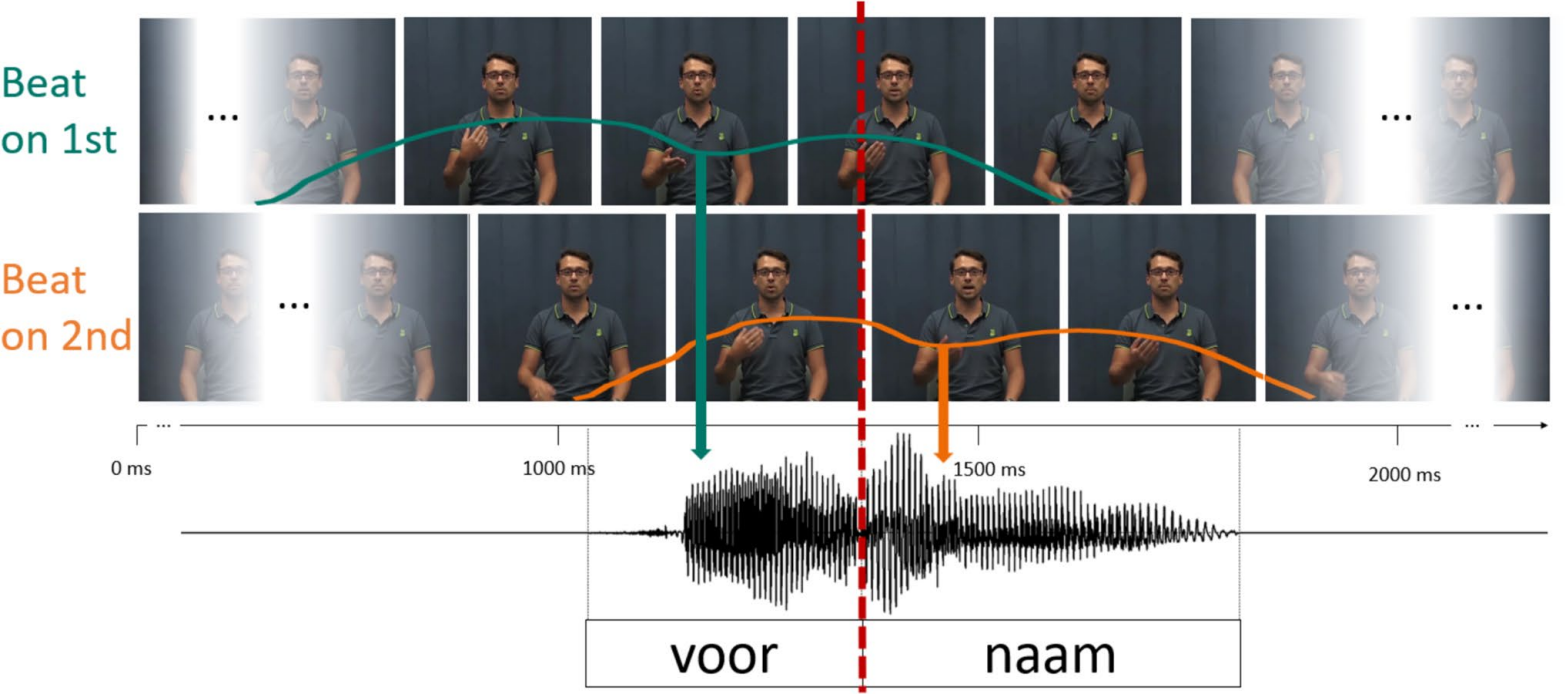
Audiovisual stimuli from Experiment 2. The apex of the beat gesture was aligned to either the first (Beat on 1 st, green) or second syllable (Beat on 2nd, orange). Colored lines show the position of the hand and thus movement of the gesture over time. Arrows indicate approximate alignment of the gesture’s apex with concurrent speech. Videos were combined with all steps of the auditory stress continuum, aligned at second syllable onset

The manipulated auditory stress continua, as described in Experiment 1, were combined with the original video recordings such that every auditory step was combined with a video with a beat gesture on the first (Beat on 1 st) and a video with a beat gesture on the second syllable (Beat on 2nd). This created our final audiovisual stimuli for use in the exposure phase (see Fig. 3). Because the duration of the audio was manipulated to make the duration cues ambiguous with regard to lexical stress, the audio was slightly misaligned with the original, unchanged video (mean = 40 ms). We aligned audio and video at second syllable onset precisely by shifting the second syllable onset of the manipulated audio to the time of the original second syllable onset. We decided to align at second syllable onset to minimize misalignment at word onset and offset. This led to a slight variation of the beat gesture alignment within each syllable, but, because of the alignment at the syllable boundary, all beat gestures were still aligned with the correct syllable.

#### Design and procedure

The experimental design was similar to that of Bosker (2022) and Experiment 1, consisting of an exposure phase and a test phase. It used the same auditory stimuli as Experiment 1. However, now in exposure, participants were exposed to the audio together with video, with the disambiguating information being beat gestures (no orthographic forms; see Table 2). Participants were again assigned to one of two conditions: the Recalibration Condition or the Generalization Condition. Within each condition participants were assigned to either the SW or WS-Bias group.

**Table 2:** Overview of the design of Experiment 2: conditions and stimuli presented

Condition	Group bias	Exposure (AV)		Test (A-only)
		Audio	Video	
Recalibration	SW bias	amb./ka:.nɔn/(Step 4)	*Beat on 1 st*	/ka:.nɔn/continuum (Steps 2–6)
		WS/ka:.nɔn/(Step 7)	*Beat on 2nd*	
	WS bias	amb./ka:.nɔn/(Step 4)	*Beat on 2nd*	
		SW/ka:.nɔn/(Step 1)	*Beat on 1 st*	
Generalization	SW bias	amb./vo:r.na:m/(Step 4)	*Beat on 1 st*	
		WS/vo:r.na:m/(Step 7)	*Beat on 2nd*	
	WS bias	amb./vo:r.na:m/(Step 4)	*Beat on 2nd*	
		SW/vo:r.na:m/(Step 1)	*Beat on 1 st*	

In the exposure phase of the Recalibration Condition, the participants in the SW-Bias group were presented with an original video of the speaker producing the word *kaNON* (i.e., stress on second syllable; WS) while making a beat gesture on the second syllable. Additionally, they were presented with an acoustically ambiguous auditory token (Step 4 from the seven-step continuum), which was disambiguated by the talker making a beat gesture on the first syllable. In contrast, participants in the WS-Bias group were presented with a video of the speaker producing clear *CAnon* (i.e., stress on the first syllable; SW) with a congruent beat gesture on the first syllable. The ambiguous auditory token was presented with a video of the talker producing a beat gesture on the second syllable. Participants in the SW-Bias group were expected to learn that the acoustic properties of the ambiguously stressed word were intended to express an SW prosodic pattern. Conversely, the WS-Bias group was expected to learn that the same ambiguous acoustic cues were intended to express a WS prosodic pattern. The exposure phase in the Generalization Condition was identical in design, but participants were presented with videos of the speaker producing a different word pair: *VOORnaam – voorNAAM.*

In total, the exposure phase had 48 trials of which 24 involved original videos and 24 involved ambiguous audios disambiguated by the temporal alignment of the beat gesture. Participants had no task in exposure and were only asked to passively watch the videos, although we emphasized that they had to pay attention to both audio and video. Participants proceeded from one trial to the next by pressing the spacebar. Each video was preceded by a fixation cross for 500 ms and then the video was played and disappeared once it stopped playing, whereupon participants were asked to press the spacebar to continue.

In the test phase, all participants were tested only on the ambiguous auditory tokens from the auditory *CAnon – kaNON* continuum (Steps 2–6), without any video. The test phase was identical to the test phase of Experiment 1 in terms of stimuli and procedure; hence it exclusively included audio-only trials (no videos).

### Results

**Fig. 4:**
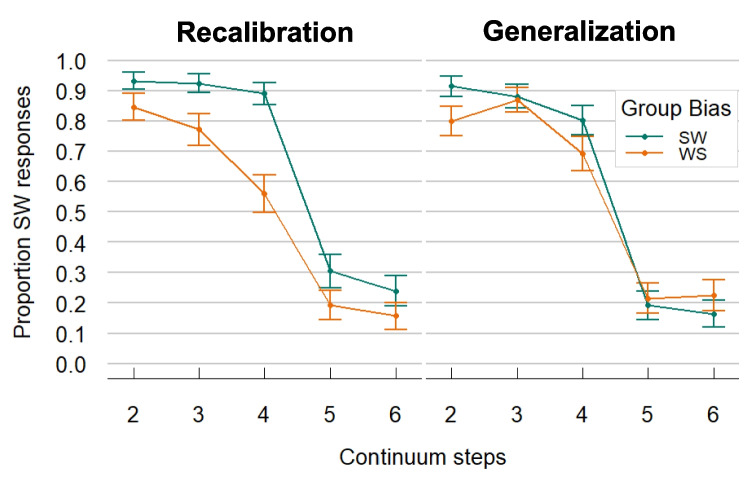
Results from Experiment 2: Recalibration driven by beat gestures. Comparison of the results of the Recalibration (**left**) and the Generalization Condition (**right**). The proportion of SW responses (i.e., stress on first syllable) is generally highest at Step 2 (most SW-like) and lowest at Step 6 (most WS-like) in both conditions. The different colored lines show if participants were in the SW (green) or WS (orange) Bias group in the exposure phase. Only in the Recalibration Condition did the Group Bias from exposure reliably affect the responses in the test phase equally across all steps. That is, participants from the SW Group Bias consistently responded more SW-like, and participants from the WS Group Bias responded more WS-like. The same effect could not reliably be found in the Generalization Condition. SW = strong–weak, stress on first syllable; WS = weak-strong, stress on second syllable. Error bars indicate the 95% confidence interval

We removed all no-response trials before analysis (*n* = 32, 0.5% of all observations). The remaining data were analyzed with GLMMs, using the same model from Experiment 1. The model revealed a significant Intercept, indicating an overall bias to give more SW than WS responses (mean proportion of SW responses = 0.57, *β* = 0.736, *SE* = 0.086, *z* = 8.609, *p* < 0.001). As expected, Step was highly significant (*β* = −2.291, *SE* = 0.2, *z* = −11.484, *p* < 0.001), reflecting our auditory stimulus manipulation. As in Experiment 1, with increasing steps (i.e., continuum becoming more WS-like), the proportion of SW responses decreased. Crucially, we found an effect of Group Bias (*β* = 0.676, *SE* = 0.144, *z* = 4.691, *p* < 0.001), which meant that participants generally gave a higher proportion of SW responses when they were in the SW-Bias group and a lower proportion of SW responses when they were in the WS-Bias group (see Fig. 4). This effect suggests successful recalibration of lexical stress perception driven by beat gesture alignment. A model with a Step*Group Bias interaction term did not improve model fit. This demonstrates an overall recalibration effect, indicating that participants generalized their Group Bias to varying degrees of ambiguity. However, there was also a significant interaction between Group Bias and Condition (*β* = −0.939, *SE* = 0.283, *z* = −3.32, *p* < 0.001). That is, the size of the Group Bias effect (i.e., the recalibration effect) was reduced in the Generalization Condition relative to the Recalibration Condition. To confirm this conclusion, the model was releveled to map the Recalibration Condition onto the intercept, and then once more with the Generalization Condition on the intercept. The releveled models confirmed that the Group Bias effect was present in the Recalibration Condition (*β* = 1.146, *SE* = 0.245, *z* = 4.673, *p* < 0.001), but was statistically not significant in the Generalization Condition (*β* = 0.206, *SE* = 0.146, *z* = 1.409, *p* = 0.159).

Additionally, we ran an exploratory analysis including only the Recalibration Condition (for analysis script and output see https://osf.io/s3p6a/). A model including Time (five batches of 15 consecutive trials) and its interaction with Bias revealed a significant Time*Bias interaction (*β* = −0.23, *SE* = 0.085, *z* = −2.715, *p* = 0.007), meaning that the recalibration effect decreased over time (see Fig. S2 in the OSM), just like in Experiment 1. Finally, while the recalibration effect was numerically larger in Experiment 2 (beat gestures) than in Experiment 1 (orthography), a post hoc comparison of the recalibration conditions across the two experiments did not yield a significant Experiment*Bias interaction (*β* = 0.615, *SE* = 0.346, *z* = 1.774, *p* = 0.076).

#### Interim discussion

Experiment 1 had demonstrated that lexical stress could be recalibrated through lexico-orthographic information (i.e., orthographic form) (Bosker, 2022). Our findings from Experiment 2 are the first to demonstrate that such recalibration can be driven by the alignment of beat gestures to speech as well. We found a Group Bias effect, indicating that participants were biased to perceive the audio-only stress continuum at test differently, depending on the disambiguating gestural information they had been presented with in exposure. Participants who had been presented with the ambiguous stimuli in exposure, disambiguated with a beat gesture aligned to the first syllable (SW), gave more SW responses at test than participants from the WS-Bias group, who had been presented with the same ambiguous auditory stimuli in exposure, but then disambiguated with a gesture aligned to the second syllable.

Again, exploratory analyses revealed a dissipation of the recalibration effect over time. That is, the effect of Group Bias was stronger at the beginning than at the end of the test block, just as in Experiment 1. The dissipation of the effect was slower than suggested by previous literature (e.g., Vroomen, van Linden, et al., 2004b), and so we were able to find stable results across a test phase of 75 trials. This is further discussed in the *General discussion*. A direct comparison of Experiments 1 and 2 revealed no statistically significant difference regarding effect size, suggesting similar recalibration effects induced by disambiguating orthography and visual beat gestures. Still, Experiment 2 replicated the recalibration finding from Experiment 1 with disambiguation information that is more prevalent in face-to-face communication (i.e., beat gestures).

In contrast, despite a numerical difference, we did not find a significant recalibration effect in the Generalization Condition, where participants were required to categorize the same words after having been exposed to segmentally different words in exposure. We could thus not replicate the generalization findings from Bosker (2022). Note that Experiments 1 and 2, just like Bosker, tested the two conditions (Recalibration vs. Generalization) between participants, which adds more noise to the data compared to a within-participants design, potentially explaining why the numerical group difference observed in the Generalization Condition in Experiment 2 was not statistically reliable. Because of this consideration, Experiment 3 used an adjusted design, where Condition was tested within participants. That is, all participants were exposed to the same word (i.e., the/ka:.nɔn/item) and tested both in the Recalibration Condition (/ka:.nɔn/) in one block and in the Generalization Condition (/vo:r.na:m/) in another. As an additional benefit of this design, the number of observations was doubled. The main predictor of interest Bias (i.e., beat gesture alignment) was still tested between-subjects, like in Experiments 1 and 2, in line with the lexically guided phonetic retuning paradigm (Norris et al., 2003).

## Experiment 3: Recalibration driven by beat gestures (with additional test phase)

### Method

#### Participants

At our target of 72 participants, the groups were not counter-balanced properly, due to a scripting error, so we decided to test an additional eight participants. This left us with the final sample of 80 native speakers of Dutch (38 female, 42 male) with a median age of 25 years (SD = 5.32, range = 18–39 years). Participants were recruited through Prolific.

#### Materials, design, and procedure

The design of this experiment was similar to Experiment 2 with the critical difference that the Recalibration and Generalization Condition were now tested within participants (see Table 3), resulting in twice as many observations per condition and participant as in Experiment 2. We reused the stimuli from Experiment 2, with the addition of Steps 2–6 from the/vo:r.na:m/continuum in the Generalization block. Group Bias remained a between-participants variable, as is common in lexically guided recalibration studies.

**Table 3:** Overview of the design of Experiment 3: conditions and stimuli presented

Group bias	Exposure (AV)		Test (A-only)	
	Audio	Video	Recalibration	Generalization
SW bias	amb./ka:.nɔn/(Step 4)	*Beat on 1 st*	/ka:.nɔn/continuum (Steps 2 −6)	/vo:r.na:m/continuum (Steps 2 −6)
	WS/ka:.nɔn/(Step 7)	*Beat on 2nd*		
WS bias	amb./ka:.nɔn/(Step 4)	*Beat on 1 st*		
	SW/ka:.nɔn/(Step 1)	*Beat on 2nd*		

The exposure phase, with two groups (SW bias vs. WS bias), was identical to the exposure phase in the Recalibration Condition of Experiment 2. Specifically, participants were presented only with *CAnon – kaNON* videos. The test phase was different from the previous experiments, as all participants were now tested in both conditions. This meant the test phase consisted of two different Blocks: the Recalibration Block and the Generalization Block. In the Recalibration Block participants were tested on the middle five Steps from the *CAnon – kaNON* continuum (Steps 2–6), which was the same word they had been exposed to in the exposure phase. In the Generalization Block they were tested on the middle five steps of the *VOORnaam – voorNAAM* continuum (Steps 2–6), to test their ability to generalize the recalibration effect to segmentally different words. All participants were exposed to the Recalibration and Generalization Condition in separate blocks, with the block order counterbalanced across participants. Each condition presented each of the five steps 15 times, resulting in a total of 75 trials per block, and 150 trials in the entire test phase.

#### Results and interim discussion

**Fig. 5:**
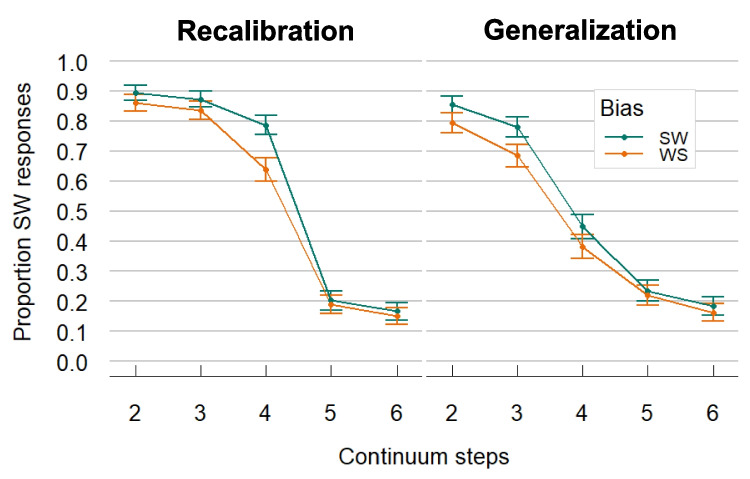
Results from Experiment 3: Recalibration driven by beat gestures (with additional test phase). Comparison of the results from the Recalibration (**left**) and the Generalization Condition (**right**). The proportion of SW responses (i.e., stress on first syllable) is generally highest at Step 2 (most SW-like) and lowest at Step 6 (most WS-like) in both conditions. The different colored lines show if participants were in the SW (green) or WS (orange) Bias group in the exposure phase. Bias group in Exposure affected the responses in the test phase in both conditions, giving evidence for recalibration. That is, people from the SW Bias group consistently responded more SW-like, and participants from the WS Bias group responded more WS-like. This recalibration effect was present in both the Recalibration Condition as well as the Generalization Condition. SW = strong–weak, stress on first syllable; WS = weak-strong, stress on second syllable. Error bars indicate the 95% confidence interval

We ran the same model as in the previous two experiments, but with the crucial difference that Condition was now a within-participant variable. Results showed a significant Intercept, reflecting a general bias to give more SW than WS responses (mean proportion SW responses = 0.52; *β* = 0.101, *SE* = 0.047, *z* = 2.15, *p* = 0.032). Again, the significant effect of Step confirmed lower proportions of SW responses for higher (i.e., more WS-like) steps (*β* = −2, *SE* = 0.17, *z* = −11.778, *p* < 0.001). Most importantly, we found a significant effect of Group Bias (see Fig. 5; *β* = 0.436, *SE* = 0.1, *z* = 4.4, *p* < 0.001). This means that participants gave more SW responses when they were in the SW-bias exposure group than when they were in the WS-bias exposure group. Another model with Step*Bias interaction did not improve model fit, suggesting no robust changes of the Group Bias effect across the different steps of the continuum. Crucially, the interaction with Condition was not significant (*β* = 0.045, *SE* = 0.131, *z* = 0.345, *p* = 0.73), suggesting that the Group Bias effect was similarly present in both conditions. Models run on either Condition as subset confirmed that the Group Bias effect was significant in the Generalization Condition (*β* = 0.393, *SE* = 0.11, *z* = 3.584, *p* < 0.001) and the Recalibration Condition (*β* = 0.456, *SE* = 0.12, *z* = 3.659, *p* < 0.001). Finally, we also observed a main effect of Condition, suggesting an overall SW-bias on the *kanon* continuum compared to the *voornaam* continuum (*β* = −0.794, *SE* = 0.1, *z* = −7.923, *p* < 0.001). An additional model with Block Order (i.e., whether participants received the Recalibration or Generalization first) did not improve model fit (see Fig. S3 in the OSM).

We also ran a post hoc model checking for changes of the Group Bias effect over time. This model included the additional predictors of Time (10 batches of 15 consecutive trials) and its interaction with Bias. Inclusion of the additional predictors improved fit and revealed a significant three-way interaction between Bias, Condition, and Time (*β* = 0.106, *SE* = 0.051, *z* = 2.09, *p* = 0.037), suggesting that the Bias effect changed over time, and did so differently across Conditions. We ran releveled models to check the effect of Bias over time for each Condition. These models included a simplified random effects structure, for reasons of convergence (only by-participant intercept and slopes for Step). In the Recalibration Condition, the Bias effect did not interact with Time (*β* = −0.048, *SE* = 0.03, *z* = −1.617, *p* = 0.106), suggesting a relatively stable effect across the experiment. In contrast, in the Generalization Condition, the Bias effect interacted with Time (*β* = 0.065, *SE* = 0.029, *z* = 2.221, *p* = 0.026), suggesting that the Bias effect *increased* over time. The results of this additional analysis, as well as plots across time, can be found in the OSM (see Fig. S4).

Thus, Experiment 3 demonstrates generalization of the recalibration effect in the perception of lexical stress. Participants who were exposed to tokens from the *CAnon – kaNON* continuum, with a beat gesture either on the first or the second syllable, responded differently in the test phase when asked to categorize a different audio-only *VOORnaam – voorNAAM* continuum. Disambiguating beat gestures on the first syllable in exposure led to more SW responses in test, while disambiguating beat gestures on the second syllable in exposure led to fewer SW responses in test, even when participants were tested on a different continuum. They thus demonstrated generalization of their recalibration to a segmentally different word.

Moreover, exploratory analyses revealed no dissipation of the recalibration effect as previously found in Experiments 1 and 2 and previous studies (e.g., Vroomen, van Linden, et al., 2004b). In the Recalibration Condition the effect appeared to remain stable across the entire experiment, and in the Generalization Condition the effect even appeared to increase over time. These findings are further discussed in the *General discussion*.

## Experiment 4: Recalibration driven by beat gestures (alternating exposure + test phases)

The previous experiments show that participants can adapt their perception of lexical stress based on lexico-orthographic information (Experiment 1) and the timing of simple hand gestures (Experiments 2 and 3). We attribute the adaptation effects to recalibration, whereby for instance the timing of a beat gesture disambiguates an ambiguous auditory stimulus. However, aside from ambiguous tokens, participants were also exposed to unambiguous clear speech tokens. Hence, our results could in principle also be explained by another type of perceptual adaptation, namely *selective adaptation* (see Eimas & Corbit, 1973). In selective adaptation, the repeated exposure of a stimulus results in adaptation *away* from the stimulus. That is, the repeated exposure to words with auditory stress on the first syllable (e.g., *CAnon*) could lead to *reduced* perception of stress on the first syllable in a following test phase (e.g., fewer *CAnon* responses). According to this selective adaptation account, our results could thus be driven by the unambiguous auditory stress cues in the clear speech tokens in exposure alone, irrespective of any contribution of beat gestures. Selective adaptation and recalibration effects can appear together (see Bertelson et al., 2003), and the design we used in the previous experiments does not allow us to disentangle the two accounts.

Another limitation of our experiments was the number of exposure and test trials we used. Previous research on visually guided recalibration has found that minimal exposure (e.g., only eight exposure trials) is sufficient to induce recalibration effects (e.g., Bertelson et al., 2003). At the same time, visually guided effects have been found to last only very briefly. For example, Vroomen et al., (2004a, 2004b) and Vroomen, van Linden et al. (2004b) found dissipation of the recalibration effect (i.e., return to baseline perception) after only six test trials. While visually guided recalibration effects have also been found in experiments with longer test phases (e.g., 84 trials in Reinisch & Mitterer, 2016), the evidence for recalibration in our previous experiments may be underestimated. In Experiment 2 we presented 75 and in Experiment 3 even 150 test trials to each participant. This may explain why we found dissipation of recalibration over the course of the test phase in Experiments 1 and 2 in our post hoc exploratory analyses.

Based on these findings and considerations, we decided to run an additional experiment^1^ that more closely followed classic visually guided recalibration studies, in which participants are presented with multiple mini-blocks including a small number of exposure trials with only ambiguous audio (i.e., eight exposure trials), followed by a small number of test trials (i.e., six test trials). Critically, Bias (i.e., Beat gesture alignment on the first vs. second syllable) was tested within-participants. That is, for every participant, in half of the mini-blocks during Exposure, the ambiguous audio was disambiguated by a beat gesture on the first syllable, while in the other half of the mini-blocks ambiguous audio was disambiguated by a beat gesture on the second syllable. The order of these Exposure + Test mini-blocks was randomized across participants. This design mitigates any potential and substantial dissipation of the recalibration effect, by close proximity between exposure and test trials as a result of the alternating mini-block design. Moreover, it also presents participants with only acoustically ambiguous trials and thus eliminates selective adaptation as an alternative explanation for the effect.

### Method

#### Power analysis

We estimated statistical power by means of Monte Carlo simulations on simulated datasets (N = 1000; Kumle et al., 2021) using GLMMs (Bates et al., 2015). We assumed an overall categorization difference of 15% between the SW and WS Bias in the Recalibration Condition (i.e., when exposure and test words are the same). This estimate was based on the averaged difference between the two Bias Groups on Steps 3, 4, and 5 in Experiments 2 and 3. With this effect size, we achieved a power of 0.80 with 24 participants in total. To be conservative and account for noisier and more complex data in the final experiment, we decided to test 40 participants in total. At this sample size, power according to our simulations was 0.95. See https://osf.io/s3p6a/ for the R code implementing this power analysis.

#### Participants

Forty native speakers of Dutch (16 female, 24 male) were recruited for this experiment through Prolific. Median age was 28 years (SD = 6.41, range = 19–40 years).

#### Materials

In this experiment we used a subset of the stimuli from the previous experiments. Specifically, we used only the video stimuli with ambiguous audio for the audiovisual exposure phase. For the audio-only test phase we selected the middle three Steps (i.e., 3, 4, 5) from the/ka:.nɔn/and/vo:r.na:m/continua. For detailed information, see earlier *Method* sections above.

#### Design and procedure

We designed this experiment similar to the classic visually guided recalibration paradigm used by Bertelson et al. (2003). Participants were presented with 32 mini-blocks, each consisting of an audiovisual exposure phase immediately followed by an audio-only test phase. Unlike previous studies (e.g., Bertelson et al., 2003; van der Zande et al., 2014; Vroomen, van Linden, et al., 2004b), our design did not include an auditory-only calibration phase preceding the experiment, serving to determine which token on a phonetic continuum was the by-participant’s most ambiguous. Instead, we used tokens that were ambiguous on the group level based on the previous experiments in order to facilitate comparison to those experiments. To limit the duration of the experimental session, we followed van der Zande et al. (2014) in testing two groups, each only receiving either/ka:.nɔn/or/vo:r.na:m/stimuli in exposure (see Table 4).

**Table 4:** Overview of the design of Experiment 4: conditions and stimuli presented. Exposure + Test mini-blocks were presented fully intermixed

	Exposure (AV)		Test (A-only)	Condition
	Audio	Video		
Group A (n = 20)	amb./ka:.nɔn/	*Beat on 1 st*	/ka:.nɔn/—continuum	Recalibration
		*Beat on 2nd*		
	amb./ka:.nɔn/	*Beat on 1 st*	/vo:r.na:m/—continuum	Generalization
		*Beat on 2nd*		
Group B (n = 20)	amb./vo:r.na:m/	*Beat on 1 st*	/ka:.nɔn/—continuum	Generalization
		*Beat on 2nd*		
	amb./vo:r.na:m/	*Beat on 1 st*	/vo:r.na:m/—continuum	Recalibration
		*Beat on 2nd*		

In each audiovisual exposure phase, participants were presented with videos of the talker producing ambiguous audio, paired with a disambiguating beat gesture on either the first or second syllable. All participants were presented with 16 mini-blocks where the audio was paired with a beat gesture on the first syllable and 16 mini-blocks where it was paired with the beat gesture on the second syllable. That is, the effect of beat gesture alignment (i.e., Bias) was tested within-participants. Within each exposure phase participants were presented with the same audiovisual stimulus eight times. The specific words participants were exposed to were counterbalanced: Half of the participants received the ambiguous token from the/ka:.nɔn/continuum and the other half received the ambiguous token from the/vo:r.na:m/continuum. Although the exposure phases did not involve any task (i.e., passive viewing), participants were explicitly instructed to pay close attention to what the talker was saying and keep looking at the screen at all times.

Each exposure phase was immediately followed by an audio-only test phase. Participants were presented with three audio-only test stimuli (steps 3, 4, and 5) twice in a random order, totaling six test trials in each phase. On every trial, a fixation cross was presented in the middle of the screen, followed by the audio-only test stimulus and two response options on either side of the screen (e.g., CAnon – kaNON). Participants were tasked to categorize the auditory stimulus as either SW (e.g., CAnon) or WS (e.g., kaNON) by pressing the left (“Z”) or right button (“M”) on their keyboard, corresponding to the left and right word on the screen, respectively. The position of SW and WS words on the screen was counter-balanced across participants. In total, participants completed 32 Exposure + Test mini-blocks. Half of the mini-blocks included Recalibration trials, while the other half included Generalization trials. In Recalibration mini-blocks, participants were tested on the same word they had been exposed to. That is, for participants who were consistently exposed to/ka:.nɔn/, the Recalibration test phases consisted of/ka:.nɔn/stimuli, and the Generalization test phase consisted of/vo:r.na:m/stimuli. Conversely, participants who were exposed to/vo:r.na:m/were presented with Recalibration test phases with/vo:r.na:m/stimuli and Generalization test phases with/ka:.nɔn/. Recalibration and Generalization Conditions were thus tested within-participants. The order of all 32 mini-blocks was fully randomized.

### Results

We used a similar GLMM model as in the experiments reported above. The model took the Categorization Response (SW coded as 1, *CAnon*; WS coded as 0; *kaNON*) as the dependent variable and the following as the predictors: Continuum Step (continuous, z-scored), Bias (categorical, deviance coded SW as 0.5 and WS as −0.5), Exposure Word (categorical, deviance coded/ka:.nɔn/as −0.5 and/vo:r.na:m/as 0.5), Condition (categorical, deviance coded Recalibration as −0.5 and Generalization as 0.5) and the interactions of Condition with the other predictors. Additionally, the model included random effects for Participants, as well as random slopes for Continuum Step and Condition (*Response* ~ Condition*(Step + *Bias* + *Exposure_word)* + *(1* + *Condition* + *Step|PP)*).

**Fig. 6:**
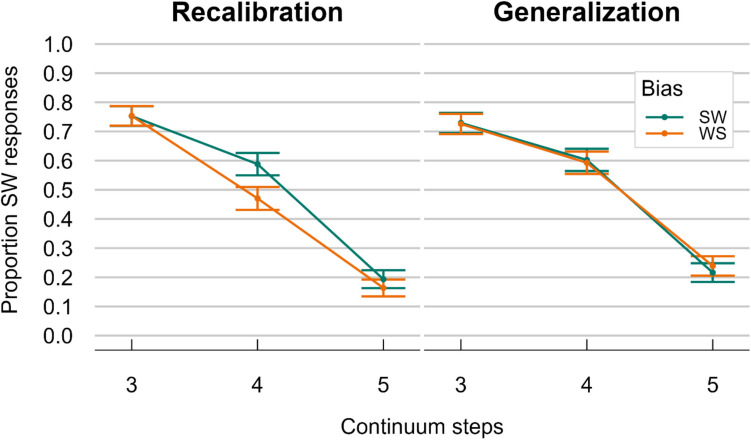
Results from Experiment 4: Recalibration driven by beat gestures (alternating Exposure + Test phases). Comparison of the results from the Recalibration (**left**) and the Generalization Condition (**right**). The proportion of SW responses (i.e., stress on first syllable) is generally highest at Step 3 (most SW-like) and lowest at Step 5 (most WS-like) in both conditions. The different colored lines show the Bias of the Beat gesture in Exposure (within-participant): SW (green) or WS (orange). Bias in Exposure affected the responses in the test phase only in the Recalibration Condition. That is, when people saw a beat gesture on the first syllable they responded more SW-like and when they saw a beat gesture on the second syllable they responded more WS-like. There was no evidence for recalibration in the Generalization Condition. SW = strong–weak, stress on first syllable; WS = weak-strong, stress on second syllable. Error bars indicate the 95% confidence interval

The model revealed a significant effect of Step, reflecting that participants gave lower proportions of SW responses on higher steps, where the auditory continuum became less SW-like (*β* = −1.192, *SE* = 0.154, *z* = −7.76, *p* < 0.001). Bias was not significant overall (*β* = 0.122, *SE* = 0.069, *z* = 1.783, *p* = 0.075), but it did interact with Condition (*β* = −0.331, *SE* = 0.137, *z* = −2.409, *p* = 0.016). Releveled models revealed that Bias was not significant in the Generalization Condition (*β* = −0.043, *SE* = 0.095, *z* = −0.454, *p* = 0.65). However, it was significant in the Recalibration condition (*β* = 0.288, *SE* = 0.1, *z* = 2.892, *p* = 0.004) (see Fig. 6). The interaction of Condition and Exposure Word was also significant (*β* = 2.308, *SE* = 0.513, *z* = 4.5, *p* < 0.001), but since these predictors are correlated it only means that proportion SW responses were generally higher for the/ka:.nɔn/continuum. The interaction of Condition and Step was also significant (*β* = 0.171, *SE* = 0.077, *z* = 2.218, *p* = 0.027), indicating that the categorization of the continua differed between the two conditions (see below).

### Interim discussion

The results of Experiment 4 confirm that beat gestures can recalibrate lexical stress perception, as audiovisual exposure to different beat gesture alignments (beat on the first or second syllable) changed subsequent audio-only perception. While the findings from Experiments 1–3 could in part have involved selective adaptation induced by exposure trials with unambiguous speech, this final experiment eliminated that possibility by only ever exposing participants to ambiguously stressed words. Again, we found no evidence for generalization of the recalibration effect to different words.

In typical visually-guided recalibration studies, participants first perform a calibration task to determine by-participant ambiguous tokens (Bertelson et al., 2003). Then every participant is exposed to speech tokens that are close to what they individually perceive as ambiguous. This provides ideal circumstances for recalibration and thus likely results in larger and more consistent recalibration effects compared to the current experiment. In contrast, for consistency with the other experiments reported here, all of our participants were exposed to the same tokens, which were ambiguous at the group level. This might have introduced more variability as individual participants could have perceived the same auditory step as more or less ambiguous and thus may have recalibrated their perception to different degrees.

Our results also show that the continua for/ka:.nɔn/and/vo:r.na:m/were perceived differently in Experiment 4, although we matched the continua based on perceptual data from an audio-only pretest (see *Methods* of Experiment 1). Therefore, any recalibration effect induced by one word may have been difficult to apply to the other word if it sounded much less ambiguous to the participant. If we had calibrated the perception of the continua per participant and per item, and the perceptual ambiguity of the two continua had been matched within participants, generalization might have been possible, as shown by Bosker (2022). Specifically, in that study the author used identical F0 contours for both continua, suggesting that generalization is possible when the suprasegmental acoustic properties of two different words are the same. Together, the results of the two studies show that generalization is likely limited, being dependent on the acoustic similarity of the two items as well as their matching in perceptual ambiguity. We note that our design is arguably closer to everyday listening conditions than earlier studies, as in naturally occurring situations of (multimodal) speech comprehension there will also be variability in the acoustics of different words as well as their perceived ambiguity.

Nevertheless, this final experiment strongly suggests that adaptation of lexical stress perception driven by audiovisual exposure to different beat gesture alignments can, at least in part, be explained by recalibration. Despite not calibrating the stimuli beforehand like previous studies have done, we were able to find a robust recalibration effect. Further studies, including such calibration to control the ambiguity of the items, may reveal even stronger evidence for recalibration and possibly generalization thereof to different words.

## General discussion

The current study investigated recalibration of lexical stress perception driven by orthography (Experiment 1) and by manual beat gestures the speaker produced while talking (Experiments 2–4). Across all four experiments, we found reliable evidence for adaptation of lexical stress perception. Experiment 4 demonstrated that the adaptation could not have been the result of selective adaptation, but must have been caused by recalibration instead. The recalibration effects emerged after mere passive exposure to the multimodal stimuli in an online testing setup without control over participants’ looking behavior or attention, and using as few as eight acoustically ambiguous trials during exposure in Experiment 4. Thus, we replicated previous recalibration findings regarding lexical stress, driven by lexico-orthographic information (Bosker, 2022), with more variable and arguably more naturalistic phonetic continua. And, most strikingly, we demonstrate recalibration of lexical stress perception driven by simple up-and-down manual beat gestures.

These results highlight the importance of beat gestures and specifically their temporal alignment to the speech signal in audiovisual speech perception. Not only articulatory visual cues (i.e., lip movements) that are causally linked to the speech signal can affect speech perception (Bertelson et al., 2003; McGurk & MacDonald, 1976), but also other visual signals such as simple up-and-down gestures produced by the hands. Here we show that these effects of gestural timing go beyond immediate perception (Bosker & Peeters, 2021) and can lead to lasting changes to perceptual representations. Remarkably, these results were obtained even though participants did not have to respond overtly to the audiovisual exposure trials. Previous studies investigating how beat gestures impact spoken word recognition (Bosker & Peeters, 2021; Bujok et al., 2025; Cos et al., 2024; Maran & Bosker, 2024; Rohrer et al., 2020) primarily relied on forced categorization of words, allowing for strategic, task-related responses (e.g., “I will always press *CAnon* when I see a beat gesture on the first syllable”). Instead, in the present study, participants viewed the videos only passively and yet these videos changed their perception of subsequent audio-only test trials. This strongly suggests that beat gestures may affect everyday spoken word recognition.

Our results are consistent with recent models of speech-gesture integration, which highlight the multimodal nature of spoken communication (Holler & Levinson, 2019; Kita & Özyürek, 2003). Further research is needed to examine whether other types of gestures can also support recalibration. Iconic gestures (e.g., Drijvers & Özyürek, 2017) and pointing gestures (Peeters, 2015; Pouw & Dixon, 2019) are also temporally aligned with the spoken signal (see McNeill, 1992; Pouw & Dixon, 2019). Yet, these gestures may offer additional cues to disambiguate the acoustic signal. Iconic gestures convey a specific meaning, and pointing gestures can direct attention, for instance when referring to a specific object. Therefore, perceptual recalibration through other types of gestures than beat gestures may be driven by temporal alignment to the speech, but possibly through other disambiguating cues as well.

Our results show how the use of beat gestures to recalibrate lexical stress perception could be a plausible explanation for suprasegmental adaptation in day-to-day communication, where beat gestures and speech co-occur frequently (in contrast to speech-disambiguating orthography that may be primarily present in subtitles). Moreover, our findings support the notion that gestures are processed and integrated automatically with speech (Kelly et al., 2010). We found recalibration effects even when participants were not instructed to pay attention to the beat gestures presented to them in exposure. More active tasks, for instance requiring comprehension of the presented words in a communicative context, might potentially lead to larger recalibration effects. However, as our experiments have shown, explicit tasks are not necessary for beat gesture integration with speech. This is in line with previous research showing effects of beat gesture alignment in more implicit tasks, like shadowing and vowel length perception (Bosker & Peeters, 2021). Evidence from exploratory analyses of Experiments 1 and 2 showed that the recalibration effects decreased over time, but in Experiment 3 with a test phase of 150 trials, we did not detect a dissipation of the recalibration effect over time. Therefore, it is unclear how long the recalibration effect typically lasts and which variables affect its persistence. These questions could be addressed in future research.

Furthermore, we tested all four experiments in an online testing setup. This limited our control over the auditory and visual display of the stimuli such as audio and video quality, video size, and audiovisual synchrony. More importantly, although participants were instructed to pay attention to the video stimuli, we had no control over their looking behavior or attention. We do not know how much the participants actually attended to the video and more specifically the beat gesture, which, in the participants’ view, was not directly relevant to the task. Still, we found consistent recalibration effects across all experiments, demonstrating both the robustness of this effect and the viability of online testing in research on audio-visual integration in speech processing.

Current models of speech adaptation (e.g., Kleinschmidt & Jaeger, 2015; Xie et al., 2023) do not address suprasegmental recalibration and/or the potential influence of visual gesture information. Still they might be able to explain our findings. Kleinschmidt and Jaeger’s (2015) Ideal Adaptor Framework proposes that the perceptual system resolves ambiguity and thus adapts by statistical inference. For example, when presented with an ambiguous sound (e.g., midway between/ba/and/da/), together with clear visual articulation (e.g., closing lips), one can infer the likely sound from prior experience (i.e., informed by the statistics about which sounds these mouth shapes usually co-occur with). In theory, the same process of inference could be used to recalibrate the perception of lexical stress. When presented with acoustically ambiguous stress cues, together with a temporally aligned visual beat gesture, one could infer that these acoustic cues convey a certain stress pattern based on the prior experience that beat gestures usually accompany stressed syllables. Still, it is unclear to what extent the underlying processes responsible for segmental and suprasegmental recalibration are the same. Moreover, it is also unclear whether these models make different predictions for beat gestures, which are only relevant in their temporal alignment to speech and do not convey any phonetic information, unlike articulatory cues that are both time-aligned and phonologically informative. As our results show that recalibration can be driven by beat gestures, models should address different sources of visual information and suprasegmental aspects of speech more specifically.

An important goal of the present study was to test for generalization of recalibration to novel and segmentally distinct words, not encountered during exposure. Bosker (2022) reported evidence for such generalization. In their study, the F0 contour of the ambiguous stimuli were identical between words, which could have facilitated generalization. Our study used more naturalistic and variable F0 contours and provides limited evidence for generalization of the recalibration effect. We did not find a generalization effect in Experiments 1, 2, and 4. However, we found a generalization effect in Experiment 3, using a design that tested Condition (Recalibration vs. Generalization) within participants. Interestingly, post-hoc exploratory analyses showed no dissipation of this effect over the course of the experiment, even though the test phase was 150 trials long. In fact, it appears that the effect grew slightly over time. Note that this finding replicates the generalization outcomes reported in Bosker (2022), but this time with more naturalistic auditory phonetic continua. Still, we have to interpret these findings with caution, as we only found evidence for generalization in one out of four experiments. Thus, our evidence suggests that generalization is possible, but less likely to occur than within-item recalibration. We speculate that generalization effects may be particularly sensitive to properties of the stimuli used, specifically the acoustic similarity of the trained and novel stimulus (see Bosker, 2022). Note, however, that acoustic similarity cannot explain why the generalization effect only surfaced in our Experiment 3, as we used the same stimuli in all experiments, albeit in different orders. That is, participants generalized either from/vo:r.na:m/to/ka:.nɔn/(Experiments 1, 2, and 4), or from/ka:.nɔn/to/vo:r.na:m/(Experiments 3 and 4). Therefore, the factors underlying generalization are still unclear and remain to be tested using different stimuli and experimental designs.

The current study examined recalibration and generalization for a small set of stimuli. Therefore, more work is needed to fully understand when recalibration and generalization do or do not happen. For instance, generalization is usually taken as evidence of phonological abstraction. According to metrical phonology, in the case of lexical stress the abstraction is a metrical structure of a whole phrase that cues relative prominence (Ladd & Arvaniti, 2023; Pierrehumbert, 1980). When perceiving speech, phonetic information cues this metrical structure. In our study, the orthography (Experiment 1) and beat gestures (Experiments 2, 3, and 4) provided additional information cueing a specific metrical structure for this speaker (i.e., acoustically ambiguous cues referring to either SW or WS). Experiment 3 showed that this cued structure can also be applied to a segmentally different disyllabic word. An interesting avenue for future research would be to investigate different and more complex metrical structures (e.g., polysyllabic words) and thus test the limits of generalization.

Another potential future topic of interest is to assess to what extent the recalibration effect, induced by beat gestures as consistently observed in the present study, is speaker-specific (Eisner & McQueen, 2005). For instance, if a listener recalibrates their perception of speaker A, will their perception of speaker B change as well, or remain unchanged? There is mixed evidence for speaker-specificity in recalibration of segmental speech representations. Some studies testing the recalibration of certain phonemes (i.e., fricatives) have found such speaker-specificity (Eisner & McQueen, 2005; Kraljic & Samuel, 2007). These studies argue that generalization to different speakers is not beneficial unless there are indications that the pronunciation variation is driven by group-level factors (e.g., demographics). Other studies, testing different phonemes (i.e., stop consonants), found generalization across speakers (e.g., Kraljic & Samuel, 2006, 2007), which could facilitate the processing of acoustic patterns that multiple talkers have in common. It is not entirely clear to what extent recalibration of suprasegmental aspects of speech, such as lexical stress, is speaker-specific. However, there is recent evidence for speaker-specific cue weighting in lexical stress (Severijnen et al., 2021), which could perhaps also apply to recalibration. Further research could explore the limits of recalibration of lexical stress and investigate the influence of beat gestures on speech comprehension more broadly.

In sum, the results of all four experiments reported here consistently show evidence for recalibration of lexical stress. Simple flicks of the hand appear to have a lasting impact on speech perception. The mere alignment of beat gestures with speech can shape our perception of lexical stress and remain effective even when beat gestures are no longer present. Hence, the temporal alignment of gestures and speech conveys important information to a listener even in passive-viewing tasks. This highlights the importance of gesture-speech integration in face-to-face communication.

## Data Availability

All experimental data, including the stimuli are publicly available on the Open Science Framework (https://osf.io/s3p6a/) under a CC-By Attribution 4.0 International license.

## Footnotes

1 As suggested by reviewers.

## Code availability

All data and scripts (R) necessary to reproduce the results reported here are publicly available on the Open Science Framework (https://osf.io/s3p6a/) under a CC-By Attribution 4.0 International license.
